# Effect of Father-Love Absence on Subjective Well-Being: The Mediating Role of Hope

**DOI:** 10.3390/bs14111097

**Published:** 2024-11-15

**Authors:** Wang Peng, Rui Hu, Yanhui Xiang

**Affiliations:** 1 Moral Culture Research Center, Hunan Normal University, Changsha 410081, China; 202280091988@hunnu.edu.cn (W.P.); roseforyoula@gmail.com (R.H.); 2 Research Center for Mental Health Education of Hunan Province, Changsha 410081, China; 3 Cognition and Human Behavior Key Laboratory of Hunan Province, Hunan Normal University, Changsha 410081, China

**Keywords:** father-love absence, the bi-factor model, subjective well-being, weekly diary method

## Abstract

Father love is recognized as an important factor in personal development. While previous research has extensively examined the adverse effects of father-love absence on subjective well-being, it is important to note that these studies often treated subjective well-being as a singular, overarching construct, potentially limiting validity and comprehensiveness compared to a bi-factor model. Consequently, this study aimed to establish a bi-factor model of subjective well-being and investigate its association with father-love absence, considering the mediating role of hope within the theoretical framework of resilience. The study employed a weekly diary method to explore the dynamic adverse effects of father-love absence on weekly subjective well-being, highlighting hope’s role in mitigating these negative impacts. Utilizing a weekly survey method with 470 participants over seven consecutive weeks, multilevel regression modeling revealed that father-love absence significantly and negatively impacts subjective well-being. Further, multilevel mediation modeling demonstrated the mediating role of hope within the resilience framework. This research has significant theoretical and practical implications for enhancing adolescent well-being through increased father involvement in parenting.

## 1. Introduction

Subjective well-being represents a prominent subject within the realm of positive psychology [1], encompassing an individual’s cognitive and affective assessment of their life, characterized by three key components: life satisfaction, positive affect, and negative affect [2]. The significance of subjective well-being extends beyond personal happiness [3], spanning crucial dimensions such as social adaptation [4,5], life meaning [6] and other facets that collectively mirror one’s mental health [7,8]. Consequently, a comprehensive exploration of the determinants influencing an individual’s subjective well-being is of paramount importance. Empirical evidence underscores a myriad of factors impacting an individual’s subjective well-being, with roots reaching back to childhood adverse experiences [9]. One noteworthy dimension of this adversity pertains to the concept of father-love absence. Notably, father-love absence does not merely denote the physical absence of a parental figure but, more intricately, signifies the emotional, behavioral, cognitive, and volitional estrangement of the father from the child during their formative years [10]. While prior research has consistently indicated that father-love absence detrimentally influences individuals’ subjective well-being [11,12], a comprehensive understanding of the underlying mechanisms and potential mitigating strategies remains incomplete.

### 1.1. The Relationship Between Father-Love Absence and Subjective Well-Being

Within the domain of subjective well-being measurement, various models have been employed in previous research, including the three-factor model, the higher-factor model, and the bi-factor model [13,14]. Notably, the bi-factor model comprises both general and specific factors. The specific factors encompass positive affect (PA), negative affect (NA), and life satisfaction (LS), representing distinct aspects of subjective well-being, while the general factor, denoted as the general subjective well-being factor (gSWB), captures the commonalities shared among the three dimensions (PA, NA, and LS). Substantial empirical evidence within the literature substantiates the validity and advantages of the bi-factor model of subjective well-being [15,16,17]. Moreover, this model has been validated across samples of different age groups in China, such as adolescents with an average age of 13 [17] and university students with an average age of 19 [18], indicating its broad applicability.

Concurrently, previous research has diligently explored the association between father-love absence and specific factors of subjective well-being, both through direct and indirect pathways. For example, investigations conducted by Flouri and Sarkadi et al. have unveiled the adverse impact of father-love absence on life satisfaction [11,19], shedding light on its influence on the cognitive dimension of subjective well-being. Additionally, prior research has established that father-love absence can greatly impact emotional well-being, leading to a decrease in positive feelings and an increase in negative emotions such as anxiety, depression, and loneliness [20,21,22], which underscores the link between father-love absence and subjective well-being on an emotional level.

Additionally, early adolescence is a crucial stage that influences individuals’ cognitive, emotional, and social development, as well as long-term well-being [23,24]. During this stage, adolescents are particularly vulnerable to the effects of father-love absence, which can bring about life uncertainties and psychological distress that profoundly impact their well-being [25]. Based on this, Hypothesis 1 of this study proposes that father-love absence affects the subjective well-being of individuals in early adolescence.

### 1.2. The Mediating Role of Hope

Resilience theory posits that resilience is the ability of individuals to achieve positive development when faced with adversity by relying on fundamental adaptive systems, which depend on the support of protective factors such as social support and positive psychological resources [26,27]. Hope, as a positive cognitive state characterized by goal orientation, the belief in one’s ability to achieve goals, and the formulation of plans to reach those goals [28], can thus be regarded as a critical protective psychological resource. The absence of fatherly love, as a form of early adversity, may diminish an individual’s sense of hope [29]. Specifically, fathers often serve as positive role models, and their involvement in a child’s upbringing fosters positive attitudes, develops problem-solving skills, and ultimately supports the cultivation of hope [30] (pp. 1–26). Conversely, the absence of fatherly love results in the lack of such role models, potentially leading to emotional, behavioral, cognitive, and volitional estrangement [10], thereby threatening the two fundamental components of hope: agency and pathways. Hope Theory further elucidates this dual structure, emphasizing that the intention to reach goals (agency) must be accompanied by the flexibility and creativity to find alternative routes when facing obstacles (pathways) [31] (pp. 257–276). Thus, father-love absence may undermine both the agency and pathways aspects of hope. On the one hand, the lack of emotional support and encouragement can result in reduced motivation and perseverance in goal pursuit, potentially decreasing the agency component of hope. On the other hand, the absence of guidance and role modeling from a father figure may restrict an individual’s ability to develop alternative strategies to cope with challenges, thus limiting the pathways component.

Additionally, from the perspective of resilience theory, hope is a significant predictor of subjective well-being. First, the theory suggests that hope is crucial for enhancing life satisfaction. As a protective psychological resource [32], hope helps alleviate the negative impact of stressful life events on life satisfaction [33], thereby increasing life satisfaction. In contrast, individuals with lower levels of hope are more likely to adopt a negative worldview [34] and develop a pessimistic thinking style [35], which can impair their ability to cope with stressful events due to insufficient psychological resources, thus reducing life satisfaction. Previous research has demonstrated a positive relationship between hope and life satisfaction. For instance, Marques et al. found that hope interventions for middle school students significantly enhanced their life satisfaction [36]. Second, according to resilience theory, hope is also a significant predictor of both positive and negative affect. The theory posits that hope serves as a positive emotional resource, contributing to positive emotions such as emotional security [37] and an optimistic outlook on the future [38]. Previous research indicates a significant positive correlation between hope and positive affect [39]. Adolescents with higher levels of hope are not only more inclined to experience positive emotions but also tend to maintain optimism in the face of adversity [40]. Conversely, adolescents with lower levels of hope are more likely to experience negative emotions, such as sadness and frustration [41]. Empirical studies further suggest that higher levels of hope in adolescents are associated with better mental health and are negatively correlated with depression and other negative emotions [42]. Moreover, comprehensive studies on the relationships between hope and various dimensions of subjective well-being have revealed significant positive correlations, suggesting that hope can enhance an individual’s life satisfaction and positive emotions while reducing negative emotions [43]. In summary, there are substantial links between hope and the specific factors of subjective well-being. Based on this, the present study proposes Hypothesis H2: Father-love absence impacts subjective well-being through the mediating role of hope in early adolescence.

### 1.3. The Present Study

Based on the review of previous studies and relevant theories, we found that while existing research on father-love absence and subjective well-being has made some progress, most studies have examined subjective well-being as immediate emotional states using cross-sectional designs. Although these cross-sectional studies provide valuable insights into the relationship between father-love absence and well-being, this design limits our understanding of how these variables change over time and fails to capture the dynamic nature of emotional states. For instance, Kesebonye and Amone-P’Olak [44] found a significant association between father involvement and the emotional health of young adult children, but due to the single time-point survey, they were unable to reveal the dynamic features of this association. Similarly, Chen & Chan [45] found a significant association between parental absence and children’s mental health, particularly in terms of emotional vulnerability and psychological distress. Cross-sectional studies have also shown that father-love absence—whether due to a lack of engagement or communication—negatively impacts adolescents’ life satisfaction [46,47].

The weekly measurement method, which assesses variables on a weekly basis, allows for timely evaluation of the relationships between the studied variables based on the obtained measurements. As an intensive longitudinal data collection approach [48] (pp. 1–8) conducted in participants’ daily environments, it is particularly well-suited for assessing relationships between state variables [49] (pp. 144–159). Compared to traditional diary methods, this approach not only enhances the reliability of results by simplifying study design and reducing participant burden but also improves the authenticity of research by capturing within-individual changes over time and individual differences in those changes [50,51]. Additionally, it offers reduced retrospective bias and higher ecological validity [49]. In summary, the present study seeks to extend previous research by utilizing the weekly diary method, grounded in the two-factor model and resilience theory, to investigate the effects of father-love absence on state subjective well-being and to explore the mediating role of state hope in this relationship. Specifically, we propose the following hypothesis: (1) Father-love absence will negatively predict subjective well-being in early adolescence; (2) Father-love absence impacts subjective well-being through the mediation of hope in early adolescence.

Building upon the tenets of resilience theory, the present study endeavors to investigate the mediating role of hope in the context of how father-love absence influences subjective well-being. By elucidating the intricate dynamics at play, this study not only advances theoretical understanding but also offers a foundational framework for enhancing the subjective well-being of individuals who have experienced father-love absence.

## 2. Methods

### 2.1. Study Design

This study employed a seven-week longitudinal diary design to investigate the relationship between father-love absence and subjective well-being among middle school students in China, while also exploring the mediating role of hope.

### 2.2. Setting

The research was conducted at a middle school in Hunan Province, China, with data collection spanning seven weeks starting from 3 June 2023. During this period, participants completed weekly paper-based questionnaires.

### 2.3. Participants

A total of 470 students (171 girls [36.4%] and 299 boys [63.6%]) from 11 classes participated in the study. Participants were recruited using cluster sampling methods. The mean age was 12.89 ± 0.48 years, with an age range of 11 to 14 years. Considering the participants were minors, permission was obtained from school administrators prior to the study. Informed consent was obtained from students who agreed to participate, and they received appropriate compensation after completing the measures each week. During data collection, there were minimal absences, and one participant transferred schools, resulting in an attrition rate of approximately 4%.

### 2.4. Variables

The primary exposure variable was father-love absence. Outcome variables included subjective well-being measures: life satisfaction, positive affect, and negative affect. The mediator variable examined was hope.

### 2.5. Data Sources and Measurement

Father-love Absence Scale (FLAS): Developed by Xiang and Zhou [10], the FLAS assesses the extent of father-love absence based on the Chinese cultural context. It contains 18 items across four subscales: emotional, cognitive, behavioral, and volition (e.g., “My father rarely considers things from my perspective”). Items are rated on a 5-point scale from 1 (“completely inconsistent”) to 5 (“completely consistent”), with higher scores indicating greater father-love absence. The scale demonstrated good reliability in this study (Cronbach’s alpha = 0.83).

Satisfaction with Life Scale (SWLS): Life satisfaction was measured using a shortened version of the SWLS [52]. To reduce participant burden, two items with the highest factor loadings were selected and appropriately adapted: (1) “In the past 5–7 days, I have been satisfied with my life”; (2) “In the past 5–7 days, my life has been close to my ideal in most aspects”. Items were rated on a 7-point Likert scale from 1 (“strongly disagree”) to 7 (“strongly agree”). The scale showed high reliability in this study (Cronbach’s alpha = 0.92).

International Positive and Negative Affect Schedule Short-Form (I-PANAS-SF): Positive and negative affect were assessed using the I-PANAS-SF [53], which includes five items each for positive and negative affect (e.g., “I have felt energized in the past 5–7 days”). Participants rated items on a 5-point scale from 1 (“strongly disagree”) to 5 (“strongly agree”). The Chinese version has demonstrated good reliability and validity [18]. In this study, Cronbach’s alpha coefficients were 0.94 for positive affect and 0.92 for negative affect.

Children’s Hope Scale (CHS): Hope was measured using a shortened version of the CHS [54]. To minimize repetition, two items with the highest factor loadings were selected and adapted: (1) “In the past 5–7 days, I felt that even if tomorrow gets worse, I still want to live my life”; (2) “In the past 5–7 days, I believed that the difficulties in my life can be solved”. Items were rated on a 6-point scale from 1 (“not at all”) to 6 (“always”). The scale demonstrated high reliability in this study (Cronbach’s alpha = 0.90).

### 2.6. Bias

To address potential common method bias due to self-report measures, Harman’s one-factor test was conducted [55]. The analysis identified ten factors with eigenvalues greater than one, and the first factor accounted for 30.00% of the variance, which is below the critical threshold of 40%. This indicates that common method bias was not a significant concern in this study.

### 2.7. Study Size

The sample size for this study was determined based on guidelines for diary studies, which recommend a minimum of 100 participants with at least five measurement occasions [56]. Our study included 470 participants assessed over seven weeks, exceeding these recommendations. To further validate the adequacy of the sample size, we conducted a sample size calculation using GPower 3.1.9.4. GPower is a widely used statistical software for conducting power analysis and sample size calculations. In this study, we performed a power analysis for an independent samples t-test, setting a two-tailed test with a significance level of α = 0.05, statistical power (1 − β) = 0.95, and an effect size of 0.5. The calculation indicated that the minimum required sample size for this study was 210, while the actual number of participants was 470, which sufficiently meets this requirement.

### 2.8. Quantitative Variables

Quantitative variables were treated as continuous measures in the analyses.

### 2.9. Statistical Methods

Descriptive Statistics and Correlation Analyses: Descriptive statistics and correlation analyses were conducted using SPSS 26.0 to provide a preliminary understanding of the relationships between key variables.

Unconditional Models: To determine the feasibility of conducting multilevel analysis, an unconditional model was constructed using Hierarchical Linear Model (HLM) 6.08. This model was employed to estimate the intraclass correlation coefficients (ICC) and reliability of the weekly measurement questions. To calculate the ICC, the weekly variance ($\sigma^{2}$) and individual variances ($\tau_{00}$) are to be substituted into the following equation:

$$\mathrm{ICC}=\frac{\tau_{00}}{\tau_{00}+\sigma^{2}}$$

According to the classification criteria of Cohen [57], ICC between 0.059 and 0.138 is considered a moderate intra-group correlation and an ICC greater than 0.138 is a high intra-group correlation coefficient.

Multilevel Regression and Mediation Analyses: Multilevel regression analyses were performed using Mplus 8.3 to examine the impact of father-love absence on subjective well-being. For the mediation analyses, we set up a multilevel 2-1-1 multiple mediation model to examine the mediating role of hope in the relationship between FLA and subjective well-being. In this study, we distinguish between between-person (between-level) and within-person (within-level) variables. Father-love absence (FLA) is treated as a between-person variable, representing differences between individuals, while hope (HOPE), life satisfaction (LS), positive affect (PA), and negative affect (NA) are treated as within-person variables, capturing individual changes over time. The multilevel model allows for the separation of variation originating from within individuals and between individuals. To assess the stability of the mediation effects, we employed a bootstrap approach by generating 5000 bootstrapped samples randomly drawn from the original dataset. This method provides bias-corrected confidence intervals for the indirect effects, enhancing the robustness of the mediation analysis.

Loss to Follow-Up: During the data collection process, four participants were absent for the first measurement, three for the second, none for the third, five were absent and one transferred for the fourth, two were absent for the fifth, one was absent for the sixth, and two were absent for the seventh. Overall, one participant was lost due to transfer, and there were 17 absences for various reasons, resulting in an overall attrition rate of approximately 4%.

Handling Missing Data: In this study, missing data were coded as “–99” and handled using the Maximum Likelihood Estimation (MLE) method. MLE is an effective statistical method under the assumption of missing data being random, as it estimates the missing values by utilizing the integrity of the available data, thereby avoiding data loss due to listwise deletion. This method not only enhances the validity of data analysis but also maintains the integrity of the sample size when dealing with incomplete data. When conducting multilevel analyses, using the MLE method can ensure the accuracy and reliability of the results. All missing data were processed in Mplus 8.3 to ensure the robustness of the analysis results.

## 3. Results

### 3.1. Preliminary Analysis

Table 1 presents the descriptive statistics for each variable, encompassing both the mean values and standard deviations. Simultaneously, it delineates the interrelationships among father-love absence, hope, life satisfaction, positive affect, negative affect and subjective well-being. The results indicate that all variables were significantly correlated.

### 3.2. Unconditional Models

In this study, the results of the unconditional model showed that ICC (Hope) = 0.29 > 0.138, ICC (Life satisfaction) = 0.38 > 0.138, ICC (Positive affect) = 0.28 > 0.138, ICC (Negative affect) = 0.33 > 0.138 (see Table 2), thus allowing for multilevel analysis.

### 3.3. The Associations of Father-Love Absence and Subjective Well-Being

In order to verify whether FLA could negatively predict Subjective well-being, we conducted a multilevel regression model with FLA as the predictor and Life satisfaction, Positive affect and Negative affect as the outcomes. The results show that FLA had significant effects on Life satisfaction (*B* = −0. 311, *p* < 0.001), Positive affect (*B* = −0.315, *p* < 0.001) and Negative affect (*B* = 0. 216, *p* < 0.001). That is, adolescents with FLA experienced lower average levels of Subjective well-being.

### 3.4. The Multilevel Multiple Mediation Analysis

**Figure 1:**
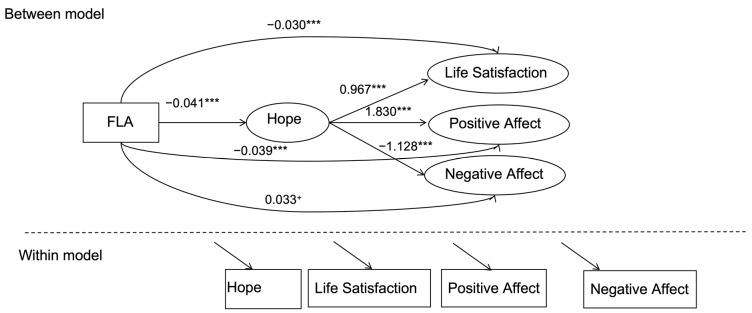
Multilevel 2-1-1 Model. Note. *** *p* < 0.001, + *p* < 0.1.

Firstly, we used the procedure recommended by Preacher et al. [58] to set up the 2-1-1 model (Figure 1). As shown in Table 3, the results indicate that FLA had significant direct effects on life satisfaction (*B* = −0.030, *p* < 0.001), positive affect (*B* = −0.039, *p* < 0.001), and negative affect (*B* = 0.033, *p* = 0.052). Additionally, the results suggested that FLA was negatively associated with hope (*B* = −0.041, *p* < 0.001), meaning that individuals experiencing father-love absence had lower levels of hope. Furthermore, hope was positively correlated with life satisfaction (*B* = 0.967, *p* < 0.001) and positive affect (*B* = 1.830, *p* < 0.001), and negatively correlated with negative affect (*B* = −1.128, *p* < 0.001).

Secondly, the mediation analysis demonstrated that hope mediated the relationships between FLA and weekly life satisfaction (95% CI = [−0.055, −0.024]), weekly positive affect (95% CI = [−0.105, −0.045]), and weekly negative affect (95% CI = [0.026, 0.066]). These findings suggest that father-love absence influences subjective well-being through its effect on individuals’ sense of hope (Table 4).

## 4. Discussion

This study embarked on a comprehensive exploration of the intricate relationship between father-love absence and subjective well-being, delving into the nuanced dimensions of affective well-being, encompassing positive and negative affect and cognitive well-being, epitomized by life satisfaction. Based on resilience theory, our findings unveiled distinctive effects of father-love absence on these facets of well-being. Furthermore, we illuminated the divergent mechanisms through which hope influences these dimensions.

### 4.1. The Relationship Between FLA and Subjective Well-Being

The findings of this study validate Hypothesis 1, providing robust evidence that father-love absence (FLA) is significantly and negatively correlated with adolescents’ subjective well-being (SWB). Specifically, the findings indicate that FLA negatively impacts life satisfaction and positive affect, while increasing the levels of negative affect. These results are consistent with previous research, which suggests that FLA has detrimental effects on various aspects of emotional and cognitive well-being [11,21].

The negative correlation between FLA and life satisfaction suggests that the absence of fatherly love is significantly associated with lower life satisfaction in adolescents. This can be attributed to the fact that FLA not only reduces objective material support [59,60] but also creates a sense of abandonment, affecting perceived subjective social support [61], thereby diminishing life satisfaction. Similarly, the reduction in positive affect due to FLA underscores the critical role of fatherly love in providing emotional support, emotional connection, and a sense of security. The lack of these emotional resources makes it more difficult for individuals to experience positive emotions [20]. Conversely, the positive correlation between FLA and negative affect indicates that adolescents experiencing FLA are more prone to feelings of sadness, anxiety, and anger. This finding aligns with previous research showing that adolescents who lack fatherly love face more challenges in daily life and have more negative experiences [25].

These findings highlight the significant direct relationships between FLA and the three key components of SWB—life satisfaction, positive affect, and negative affect—emphasizing the crucial role of father-love absence in shaping individual subjective well-being.

### 4.2. Mediating Factors in the Relation Between FLA and Subjective Well-Being

Our mediation analysis supported Hypothesis 2, confirming the crucial mediating role of hope in the relationship between father-love absence and subjective well-being during early adolescence. Hope significantly moderated the relationships between FLA and life satisfaction (LS), positive affect (PA), and negative affect (NA).

First, with regard to life satisfaction (LS), resilience theory posits that hope serves as a protective mechanism, playing a critical role in fostering subjective well-being when individuals face stress and adversity. The outcomes of our study not only corroborate the association between hope and father-love absence but also highlight the multifaceted factors that shape an individual’s hope throughout their developmental journey [62]. Empirical evidence indicates that father-love absence is linked to heightened stress and uncertainty during formative years [63], significantly impacting the development of hope [64]. Additionally, this study aligns with prior research showing that individuals with lower levels of hope tend to adopt more pessimistic mindsets and coping strategies when dealing with stress and adversity [65]. This inclination towards negative approaches leads to unfavorable outcomes, culminating in diminished life satisfaction [66]. In essence, our study underscores that individuals experiencing father-love absence have lower life satisfaction, primarily due to their diminished sense of hope.

Secondly, regarding positive affect (PA) and negative affect (NA), resilience theory suggests that hope also functions as a cognitive protective factor, fostering a positive mindset in individuals. Those with a high sense of hope perceive challenges and obstacles as opportunities for success rather than as stressors. This cognitive flexibility and problem-solving tendency reduce the intensity of negative affect, predisposing individuals to experience more positive emotions [67]. Moreover, Lloyd’s study found that high levels of hope protect individuals from the detrimental effects of depression, suggesting that hope has a compensatory function—predicting a decrease in psychological distress and an increase in positive mood. In contrast, individuals with low levels of hope are more cognitively inclined to adopt negative attributions [68]. They are more likely to focus on their shortcomings and objective situational obstacles, leading to a generalized sense of helplessness and a greater tendency to experience negative emotions such as depression [69]. In summary, father-love absence further influences positive and negative affect by impacting hope.

In conclusion, this study confirmed the mediating role of hope in the relationship between father-love absence and subjective well-being, including life satisfaction, positive affect, and negative affect. Father-love absence, by weakening the sense of hope, decreases life satisfaction, increases negative emotions, and reduces positive emotions. Hope, as a cognitive protective mechanism, helps mitigate these negative effects and enhances individuals’ subjective well-being.

### 4.3. Limitations and Future Research

This study explores the impact of father-love absence on adolescents’ subjective well-being and examines the mediating role of hope. However, the findings have certain limitations. While the study focuses on the effects of father-love absence on adolescents, it does not control for other variables related to the broader adolescent living environment, such as cultural context, family structure, and peer relationships. Future research could incorporate these variables to clarify the unique mechanisms among them.

### 4.4. Implications

Our study yields theoretical and practical insights into the enhancement of subjective well-being within populations grappling with father-love absence. First and foremost, our research underscores the negative association between father-love absence and subjective well-being, as well as the pivotal mediating role of hope, shedding light on a crucial mechanism through which father-love absence influences adolescents’ subjective well-being. This novel perspective advances our understanding of the intricate interplay between father-love absence and subjective well-being and provides a theoretical basis for elucidating the underlying dynamics. Furthermore, the extant literature on father-love absence predominantly emphasizes its deleterious consequences. By introducing hope as a potential mediating variable, our study enriches the landscape of positive psychology research by introducing positive psychological factors that can ameliorate the negative impacts of father-love absence. This paradigm shift not only complements existing scholarship but also broadens the scope of inquiry within the field. Moreover, our investigation has the potential to inform interventions and support programs tailored to individuals contending with father-love absence. Hope emerges as a malleable cognitive attribute that can be nurtured and cultivated in these individuals. By instilling hope, interventions can empower individuals to cultivate a more positive outlook on life, navigate the challenges and vicissitudes associated with paternal absence, and consequently enhance their subjective well-being and overall resilience.

## 5. Conclusions

This study confirmed the positive correlation between early father-love absence and adolescents’ subjective well-being. Further multilevel mediation analysis demonstrated the mediating role of hope in this relationship. These findings not only enrich the research on father-love absence but also extend the dual-factor model and resilience theory. They indicate that early adverse environments can affect adolescents’ subjective well-being and have a lasting negative impact by influencing their sense of hope, underscoring significant theoretical implications. Moreover, this study holds substantial practical significance, offering new theoretical and empirical perspectives on enhancing adolescents’ subjective well-being and providing effective recommendations for improving their well-being levels.

## Figures and Tables

**Table 1:** Descriptive statistics and correlations for all variables.

	*M*	*SD*	1	2	3	4	5
Between level							
1. FLA	41.22	11.67	1				
Within level							
2. Hope	9.75	2.23	−0.215 ***	1			
3. Life satisfaction	9.48	3.29	−0.246 ***	0.558 ***	1		
4. Positive affect	19.00	4.96	−0.268 ***	0.703 ***	0.684 ***	1	
5. Negative affect	11.46	5.27	0.177 ***	−0.395 ***	−0.449 ***	−0.433 ***	1

Note. *** *p* < 0.001.

**Table 2:** Weekly inspection of measurement items.

Item	*M*	$\tau_{00}$	$\sigma^{2}$	ICC	Reliability
1. Hope	9.75	1.44	3.56	0.29	0.95
2. Life satisfaction	9.48	4.07	6.78	0.38	0.92
3. Positive affect	19.00	6.82	17.82	0.28	0.95
4. Negative affect	11.46	9.27	18.49	0.33	0.93

**Table 3:** Variables on weekly Subjective well-being.

	Weekly Life Satisfaction			Weekly Positive Affect			Weekly Negative Affect		
	*B*	*SE*	*p*	*B*	*SE*	*p*	*B*	*SE*	*p*
FLA	−0.030 ***	0.008	<0.001	−0.039 ***	0.010	<0.001	0.033 +	0.017	0.052
Hope	0.967 ***	0.054	<0.001	1.830 ***	0.072	<0.001	−1.128 ***	0.130	<0.001

Note. FLA is father-love absence. *** *p* < 0.001; + *p* < 0.1.

**Table 4:** Standardized indirect effect and 95% confidence interval.

Pathways	Estimate	Lower	Upper
FLA→Hope→Life satisfaction	−0.040	−0.055	−0.024
FLA→Hope→Positive affect	−0.075	−0.105	−0.045
FLA→Hope→Negative affect	0.046	0.026	0.066

## Data Availability

The data in this study are available from the corresponding authors upon reasonable request.
